# 4-[(4-Chloro­phen­yl)(hy­droxy)methyl­idene]isochromane-1,3-dione

**DOI:** 10.1107/S160053681104829X

**Published:** 2011-11-19

**Authors:** Akoun Abou, Abdoulaye Djandé, Bintou Sessouma, Adama Saba, Rita Kakou-Yao

**Affiliations:** a Laboratoire de Cristallographie et Physique Moléculaire, UFR SSMT, Université de Cocody 22 BP 582 Abidjan 22, Côte d’Ivoire; b Laboratoire de Chimie Bio-organique et Phytochimie, Université de Ouagadougou 03 BP 7021 Ouagadougou 03, Burkina Faso

## Abstract

In the title compound, C_16_H_9_ClO_4_, the six-membered heterocyclic ring adopts a screw-boat conformation. The benzene rings are oriented to each other at a dihedral angle of 59.26 (9)°. The mol­ecular structure exhibits a ring motif, *viz. S*(6), owing to an intra­molecular O—H⋯O hydrogen bond. The presence of C—H⋯O contacts generates an infinite chain along [001]. Also present are π–π stacking inter­actions between neighbouring isochromanedione benzene rings [centroid–centroid distance = 3.746 (1) Å], and C—O⋯π inter­actions [O⋯centroid = 3.934 (2) Å].

## Related literature

For the biological activity of isochromanones, see: Bianchi *et al.* (2004 ▶); Buntin *et al.* (2008 ▶). For π–π stacking inter­actions, see: Janiak (2000 ▶). For hydrogen-bond motifs, see: Bernstein *et al.* (1995 ▶). For puckering parameters, see: Cremer & Pople (1975 ▶).
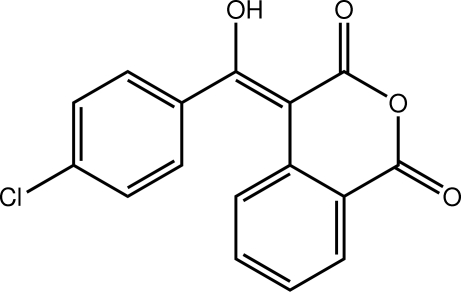


## Experimental

### 

#### Crystal data

C_16_H_9_ClO_4_
*M* *_r_* = 300.68
Monoclinic, 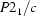

*a* = 15.4973 (4) Å
*b* = 5.9631 (1) Å
*c* = 14.4526 (3) Å
β = 102.661 (1)°
*V* = 1303.12 (5) Å^3^
*Z* = 4
Mo *K*α radiation
μ = 0.31 mm^−1^
*T* = 298 K
0.40 × 0.30 × 0.20 mm

#### Data collection

Nonius KappaCCD diffractometer
12213 measured reflections
3248 independent reflections
2693 reflections with *I* > 2σ(*I*)
*R*_int_ = 0.037

#### Refinement

*R*[*F* ^2^ > 2σ(*F* ^2^)] = 0.053
*wR*(*F* ^2^) = 0.163
*S* = 1.08
3248 reflections
191 parameters
H-atom parameters constrained
Δρ_max_ = 0.25 e Å^−3^
Δρ_min_ = −0.31 e Å^−3^

### 

Data collection: *COLLECT* (Nonius, 2001 ▶); cell refinement: *DENZO*/*SCALEPACK* (Otwinowski & Minor, 1997 ▶); data reduction: *DENZO*/*SCALEPACK*; program(s) used to solve structure: *SIR92* (Altomare *et al.*, 1994 ▶); program(s) used to refine structure: *SHELXL97* (Sheldrick, 2008 ▶); molecular graphics: *PLATON* (Spek, 2009 ▶); software used to prepare material for publication: *SHELXL97*, *publCIF* (Westrip, 2010 ▶) and *WinGX* (Farrugia, 1999 ▶).

## Supplementary Material

Crystal structure: contains datablock(s) I, global. DOI: 10.1107/S160053681104829X/tk5016sup1.cif

Structure factors: contains datablock(s) I. DOI: 10.1107/S160053681104829X/tk5016Isup2.hkl

Supplementary material file. DOI: 10.1107/S160053681104829X/tk5016Isup3.cml

Additional supplementary materials:  crystallographic information; 3D view; checkCIF report

## Figures and Tables

**Table 1:** Hydrogen-bond geometry (Å, °)

*D*—H⋯*A*	*D*—H	H⋯*A*	*D*⋯*A*	*D*—H⋯*A*
O14—H14⋯O12	0.82	1.76	2.492 (2)	148
C3—H3⋯O11^i^	0.93	2.56	3.288 (2)	136

Symmetry code: (i) 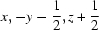.

**Fig. 1.**
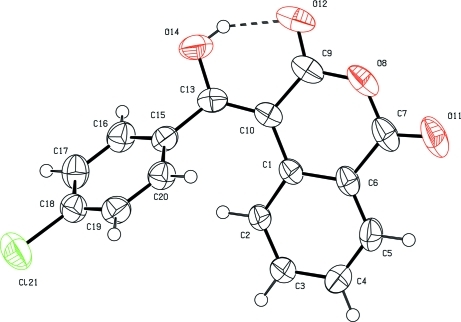
The molecular structure of (I) showing the atomic labeling scheme with displacement ellipsoids drawn at the 50% probability level. H atoms are shown as spheres of arbitrary radius. Dashed lines indicate an hydrogen bond.

**Fig. 2.**
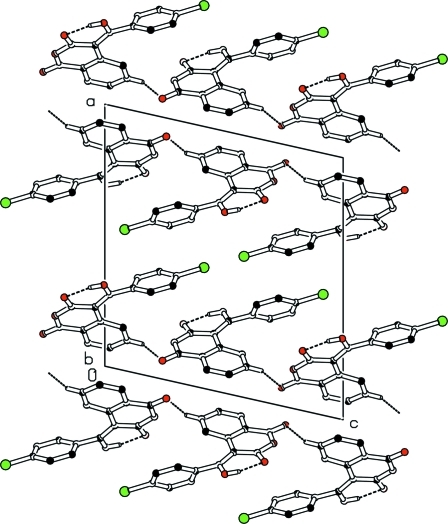
Crystal packing, viewed down the b axis, showing parallel chains along the c direction. Dashed lines indicate C—H···O contacts. H atoms not involved in hydrogen bonds have been omitted for clarity.

**Fig. 3.**
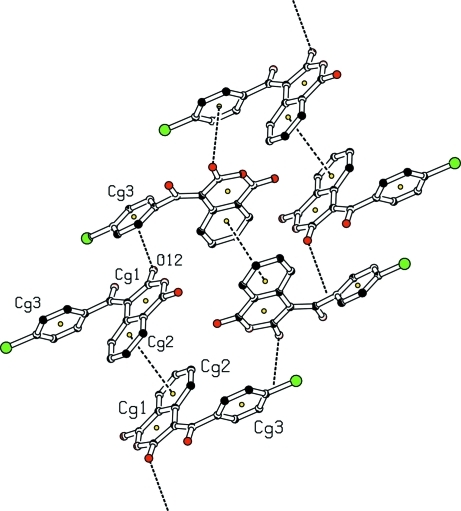
A view of the crystal packing, showing C—O···π and π–π stacking interactions (dashed lines). The yellow dots are centroids of rings. H atoms have been omitted for clarity.

## supplementary crystallographic information

### Comment

The title molecule is related to isochromanone derivatives that are generally known as regulators of plant growth (Bianchi *et al.*, 2004). Depending on their chemical structure and concentration they can act either as inhibitors or stimulators of these processes. Some substituted isochromanones isolated from myxobacteria strains were introduced as anti-fungal agents (Buntin *et al.*, 2008).

The structure of the title compound (I) (Fig. 1) consists of two planar benzene rings with the maximum deviations from the best planes of 0.035 (2) Å for atom C1 (benzene ring C1—C6) and ±0.007 (2) Å for atoms C15 and C16 (benzene ring C15—C20). An *S*(6) ring motif (Bernstein *et al.*, 1995), arises from an intramolecular O—H···O hydrogen bond to generate a planar pseudo six-membered ring (maximum deviation from planarity being 0.059 (2) Å for atom C13) to result in a tricyclic ring system (Fig. 1). The dihedral angles between two benzene rings is 59.26 (9) and that between the pseudo six-membered ring and benzene ring C1—C6 is 13.65 (9) °. The heterocyclic ring C1/C6/C7/O8/C9/C10 adopts a screw-boat conformation as judged from the puckering parameters (Cremer & Pople, 1975): Q = 0.0952 (19) Å, θ = 67.5 (11)° and φ = 228.4 (12)°. Furthermore, intermolecular C—H···O contacts (Table 1) link molecules into infinite chains through along [001] (Fig. 2).

The supramolecular aggregation is completed by the presence of C—O···π interactions (O12···*Cg*3[*x*,1/2 - *y*,-1/2 + *z*] = 3.934 (2) Å, C9—O12···*Cg*3 = 83.48 (12)°, where *Cg3* is the centroid of the benzene ring C15—C20, and π–π stacking between two parallel isochromanedione-benzene C1—C6 rings; in the latter, the centroid···centroid distance, (*Cg*2···*Cg*2 (-*x*,-*y*,-*z*) of 3.746 (1) Å), is less than 3.8 Å, the maximum regarded as relevant for π–π interactions (Janiak, 2000) (Fig.3).

### Experimental

To a solution of 4-chlorobenzoyl chloride (4.10 ^-2^ mol) in dry tetrahydrofuran (150 ml ), was added dried triethylamine (0.12 mol) and homophthalic anhydride (4.10 ^-2^ mol) in small portions over 30 min. The mixture was then refluxed for 3 h and poured in 300 ml of chloroform. The solution was acidified with dilute hydrochloric acid until the pH was 2 - 3. The organic layer was extracted, washed with water, dried over MgSO_4_ and the solvent removed. The crude product was recrystallized from a chloroform-hexane (1/1, *v*/*v*) mixture. Yellow crystals were obtained in a good yield: 90%; *M*.pt. 432–433 K.

### Refinement

H atoms were placed in calculated positions (O—H = 0.82 Å and C—H = 0.93 Å) and refined using a riding model approximation with *U*_iso_(H) constrained to 1.2 (aromatic) or 1.5 (O—H) times *U*_eq_ of the respective parent atom.

### Crystal data

**Table:**

C_16_H_9_ClO_4_	*F*(000) = 616
*M**_r_* = 300.68	*D*_x_ = 1.533 Mg m^−^^3^
Monoclinic, *P*2_1_/*c*	Melting point = 432–433 K
Hall symbol: -P 2ybc	Mo *K*α radiation, λ = 0.71073 Å
*a* = 15.4973 (4) Å	Cell parameters from 12213 reflections
*b* = 5.9631 (1) Å	θ = 1.4–29.0°
*c* = 14.4526 (3) Å	µ = 0.31 mm^−^^1^
β = 102.661 (1)°	*T* = 298 K
*V* = 1303.12 (5) Å^3^	Prism, yellow
*Z* = 4	0.40 × 0.30 × 0.20 mm

### Data collection

**Table:**

Nonius KappaCCD diffractometer	2693 reflections with *I* > 2σ(*I*)
Radiation source: fine-focus sealed tube	*R*_int_ = 0.037
graphite	θ_max_ = 29.0°, θ_min_ = 1.4°
φ and ω scans	*h* = −20→20
12213 measured reflections	*k* = −7→7
3248 independent reflections	*l* = −19→19

### Refinement

**Table:**

Refinement on *F*^2^	Primary atom site location: structure-invariant direct methods
Least-squares matrix: full	Secondary atom site location: difference Fourier map
*R*[*F*^2^ > 2σ(*F*^2^)] = 0.053	Hydrogen site location: inferred from neighbouring sites
*wR*(*F*^2^) = 0.163	H-atom parameters constrained
*S* = 1.08	*w* = 1/[σ^2^(*F*_o_^2^) + (0.085*P*)^2^ + 0.4082*P*] where *P* = (*F*_o_^2^ + 2*F*_c_^2^)/3
3248 reflections	(Δ/σ)_max_ < 0.001
191 parameters	Δρ_max_ = 0.25 e Å^−^^3^
0 restraints	Δρ_min_ = −0.31 e Å^−^^3^
36 constraints	

### Special details

**Table:**

Geometry. All s.u.'s (except the s.u. in the dihedral angle between two l.s. planes) are estimated using the full covariance matrix. The cell s.u.'s are taken into account individually in the estimation of s.u.'s in distances, angles and torsion angles; correlations between s.u.'s in cell parameters are only used when they are defined by crystal symmetry. An approximate (isotropic) treatment of cell s.u.'s is used for estimating s.u.'s involving l.s. planes.
Refinement. Refinement of *F*^2^ against ALL reflections. The weighted *R*-factor *wR* and goodness of fit *S* are based on *F*^2^, conventional *R*-factors *R* are based on *F*, with *F* set to zero for negative *F*^2^. The threshold expression of *F*^2^ > 2σ(*F*^2^) is used only for calculating *R*-factors(gt) *etc*. and is not relevant to the choice of reflections for refinement. *R*-factors based on *F*^2^ are statistically about twice as large as those based on *F*, and *R*- factors based on ALL data will be even larger.

### Fractional atomic coordinates and isotropic or equivalent isotropic displacement parameters (Å^2^)

**Table:**

	*x*	*y*	*z*	*U*_iso_*/*U*_eq_	
Cl21	0.46136 (4)	0.22385 (12)	0.41605 (4)	0.0768 (3)	
O8	0.15001 (11)	0.2734 (3)	−0.20791 (9)	0.0600 (4)	
C1	0.16846 (10)	0.1431 (3)	−0.01749 (10)	0.0355 (3)	
C6	0.11986 (11)	0.0094 (3)	−0.09040 (10)	0.0412 (4)	
O12	0.22812 (11)	0.5783 (3)	−0.16990 (11)	0.0688 (5)	
C2	0.16289 (11)	0.0881 (3)	0.07551 (10)	0.0391 (4)	
H2	0.1898	0.1804	0.1254	0.047*	
C10	0.21737 (11)	0.3377 (3)	−0.04175 (11)	0.0401 (4)	
O14	0.30865 (10)	0.6583 (2)	−0.00417 (11)	0.0631 (4)	
H14	0.2881	0.6813	−0.0606	0.095*	
C7	0.11070 (14)	0.0730 (3)	−0.18995 (12)	0.0525 (5)	
C13	0.27868 (11)	0.4635 (3)	0.02079 (13)	0.0437 (4)	
C19	0.40760 (12)	0.1419 (3)	0.22901 (14)	0.0479 (4)	
H19	0.4368	0.0053	0.2416	0.057*	
O11	0.07046 (13)	−0.0255 (3)	−0.25801 (10)	0.0769 (5)	
C5	0.07423 (13)	−0.1807 (3)	−0.07083 (13)	0.0507 (4)	
H5	0.0429	−0.2681	−0.1203	0.061*	
C4	0.07567 (13)	−0.2382 (3)	0.02135 (15)	0.0521 (5)	
H4	0.0482	−0.3690	0.0349	0.062*	
C16	0.32267 (13)	0.5515 (3)	0.19262 (14)	0.0504 (4)	
H16	0.2949	0.6900	0.1802	0.060*	
C15	0.32154 (11)	0.4012 (3)	0.11887 (12)	0.0413 (4)	
C20	0.36492 (12)	0.1967 (3)	0.13808 (13)	0.0452 (4)	
H20	0.3651	0.0961	0.0889	0.054*	
C17	0.36473 (13)	0.4967 (3)	0.28416 (14)	0.0547 (5)	
H17	0.3648	0.5966	0.3336	0.066*	
C3	0.11838 (12)	−0.1000 (3)	0.09398 (12)	0.0454 (4)	
H3	0.1169	−0.1350	0.1563	0.054*	
C18	0.40658 (12)	0.2924 (3)	0.30156 (13)	0.0484 (4)	
C9	0.19987 (13)	0.4065 (3)	−0.13974 (12)	0.0499 (4)	

### Atomic displacement parameters (Å^2^)

**Table:**

	*U*^11^	*U*^22^	*U*^33^	*U*^12^	*U*^13^	*U*^23^
Cl21	0.0704 (4)	0.1024 (5)	0.0503 (3)	0.0049 (3)	−0.0029 (3)	0.0100 (3)
O8	0.0778 (10)	0.0730 (10)	0.0291 (6)	0.0145 (7)	0.0118 (6)	0.0079 (6)
C1	0.0389 (7)	0.0382 (8)	0.0293 (7)	0.0059 (6)	0.0070 (6)	−0.0002 (5)
C6	0.0459 (8)	0.0452 (9)	0.0303 (7)	0.0107 (7)	0.0032 (6)	−0.0060 (6)
O12	0.0821 (11)	0.0719 (10)	0.0570 (8)	0.0075 (8)	0.0251 (7)	0.0298 (7)
C2	0.0427 (8)	0.0448 (9)	0.0290 (7)	−0.0061 (6)	0.0059 (6)	−0.0030 (6)
C10	0.0452 (8)	0.0422 (8)	0.0347 (7)	0.0065 (6)	0.0124 (6)	0.0057 (6)
O14	0.0663 (9)	0.0489 (8)	0.0720 (10)	−0.0103 (7)	0.0107 (7)	0.0188 (7)
C7	0.0646 (11)	0.0585 (11)	0.0313 (8)	0.0215 (9)	0.0034 (7)	−0.0057 (7)
C13	0.0444 (8)	0.0379 (8)	0.0508 (9)	0.0024 (6)	0.0147 (7)	0.0074 (7)
C19	0.0424 (8)	0.0427 (9)	0.0569 (10)	0.0037 (7)	0.0075 (7)	0.0054 (7)
O11	0.1078 (13)	0.0786 (11)	0.0337 (7)	0.0195 (9)	−0.0076 (7)	−0.0149 (7)
C5	0.0531 (10)	0.0472 (10)	0.0456 (9)	0.0004 (8)	−0.0030 (7)	−0.0133 (7)
C4	0.0534 (10)	0.0446 (10)	0.0550 (11)	−0.0112 (8)	0.0052 (8)	−0.0023 (8)
C16	0.0512 (10)	0.0372 (9)	0.0597 (11)	0.0044 (7)	0.0056 (8)	−0.0054 (7)
C15	0.0384 (8)	0.0365 (8)	0.0483 (9)	−0.0032 (6)	0.0081 (7)	0.0014 (6)
C20	0.0472 (9)	0.0387 (9)	0.0493 (9)	0.0025 (7)	0.0097 (7)	−0.0033 (7)
C17	0.0549 (11)	0.0544 (11)	0.0523 (10)	0.0022 (8)	0.0068 (8)	−0.0133 (8)
C3	0.0476 (9)	0.0502 (10)	0.0376 (8)	−0.0064 (7)	0.0074 (7)	0.0035 (7)
C18	0.0384 (8)	0.0576 (11)	0.0473 (9)	−0.0039 (7)	0.0054 (7)	0.0028 (8)
C9	0.0552 (10)	0.0583 (11)	0.0392 (8)	0.0153 (8)	0.0171 (7)	0.0120 (7)

### Geometric parameters (Å, °)

**Table:**

Cl21—C18	1.7349 (19)	C13—C15	1.475 (2)
O8—C9	1.365 (3)	C19—C20	1.375 (3)
O8—C7	1.391 (3)	C19—C18	1.383 (3)
C1—C6	1.402 (2)	C19—H19	0.9300
C1—C2	1.405 (2)	C5—C4	1.371 (3)
C1—C10	1.469 (2)	C5—H5	0.9300
C6—C5	1.397 (3)	C4—C3	1.384 (3)
C6—C7	1.465 (2)	C4—H4	0.9300
O12—C9	1.231 (2)	C16—C17	1.380 (3)
C2—C3	1.373 (2)	C16—C15	1.390 (2)
C2—H2	0.9300	C16—H16	0.9300
C10—C13	1.381 (2)	C15—C20	1.390 (2)
C10—C9	1.442 (2)	C20—H20	0.9300
O14—C13	1.330 (2)	C17—C18	1.377 (3)
O14—H14	0.8200	C17—H17	0.9300
C7—O11	1.197 (2)	C3—H3	0.9300
			
C9—O8—C7	124.50 (14)	C6—C5—H5	120.1
C6—C1—C2	116.77 (15)	C5—C4—C3	119.43 (17)
C6—C1—C10	119.35 (14)	C5—C4—H4	120.3
C2—C1—C10	123.78 (14)	C3—C4—H4	120.3
C5—C6—C1	121.45 (15)	C17—C16—C15	120.40 (17)
C5—C6—C7	117.68 (16)	C17—C16—H16	119.8
C1—C6—C7	120.75 (17)	C15—C16—H16	119.8
C3—C2—C1	121.06 (15)	C16—C15—C20	119.22 (16)
C3—C2—H2	119.5	C16—C15—C13	120.09 (16)
C1—C2—H2	119.5	C20—C15—C13	120.61 (16)
C13—C10—C9	116.26 (16)	C19—C20—C15	120.63 (17)
C13—C10—C1	126.15 (14)	C19—C20—H20	119.7
C9—C10—C1	117.58 (15)	C15—C20—H20	119.7
C13—O14—H14	109.5	C18—C17—C16	119.31 (18)
O11—C7—O8	116.02 (18)	C18—C17—H17	120.3
O11—C7—C6	127.0 (2)	C16—C17—H17	120.3
O8—C7—C6	116.98 (16)	C2—C3—C4	121.07 (16)
O14—C13—C10	121.90 (16)	C2—C3—H3	119.5
O14—C13—C15	111.74 (16)	C4—C3—H3	119.5
C10—C13—C15	126.31 (15)	C17—C18—C19	121.25 (18)
C20—C19—C18	119.17 (17)	C17—C18—Cl21	119.68 (16)
C20—C19—H19	120.4	C19—C18—Cl21	119.05 (15)
C18—C19—H19	120.4	O12—C9—O8	114.66 (16)
C4—C5—C6	119.89 (16)	O12—C9—C10	125.43 (19)
C4—C5—H5	120.1	O8—C9—C10	119.89 (17)
			
C2—C1—C6—C5	−5.3 (2)	C17—C16—C15—C20	−1.4 (3)
C10—C1—C6—C5	178.26 (15)	C17—C16—C15—C13	−178.23 (17)
C2—C1—C6—C7	170.79 (15)	O14—C13—C15—C16	52.6 (2)
C10—C1—C6—C7	−5.7 (2)	C10—C13—C15—C16	−129.9 (2)
C6—C1—C2—C3	5.7 (2)	O14—C13—C15—C20	−124.18 (18)
C10—C1—C2—C3	−177.98 (15)	C10—C13—C15—C20	53.3 (2)
C6—C1—C10—C13	−169.15 (16)	C18—C19—C20—C15	0.3 (3)
C2—C1—C10—C13	14.6 (3)	C16—C15—C20—C19	0.8 (3)
C6—C1—C10—C9	11.3 (2)	C13—C15—C20—C19	177.65 (16)
C2—C1—C10—C9	−164.88 (16)	C15—C16—C17—C18	0.8 (3)
C9—O8—C7—O11	−178.66 (18)	C1—C2—C3—C4	−1.7 (3)
C9—O8—C7—C6	3.6 (3)	C5—C4—C3—C2	−3.1 (3)
C5—C6—C7—O11	−3.0 (3)	C16—C17—C18—C19	0.3 (3)
C1—C6—C7—O11	−179.20 (19)	C16—C17—C18—Cl21	178.84 (15)
C5—C6—C7—O8	174.43 (15)	C20—C19—C18—C17	−0.9 (3)
C1—C6—C7—O8	−1.8 (2)	C20—C19—C18—Cl21	−179.41 (14)
C9—C10—C13—O14	11.1 (3)	C7—O8—C9—O12	−179.24 (16)
C1—C10—C13—O14	−168.40 (16)	C7—O8—C9—C10	2.3 (3)
C9—C10—C13—C15	−166.14 (16)	C13—C10—C9—O12	−7.6 (3)
C1—C10—C13—C15	14.3 (3)	C1—C10—C9—O12	172.00 (17)
C1—C6—C5—C4	0.7 (3)	C13—C10—C9—O8	170.70 (16)
C7—C6—C5—C4	−175.45 (18)	C1—C10—C9—O8	−9.7 (2)
C6—C5—C4—C3	3.6 (3)		

### Hydrogen-bond geometry (Å, °)

**Table:**

*D*—H···*A*	*D*—H	H···*A*	*D*···*A*	*D*—H···*A*
O14—H14···O12	0.82	1.76	2.492 (2)	148
C3—H3···O11^i^	0.93	2.56	3.288 (2)	136

Symmetry codes: (i) *x*, −*y*−1/2, *z*+1/2.
